# The Improved Method for Determination of Orotic Acid in Milk by Ultra-Fast Liquid Chromatography with Optimized Photodiode Array Detection

**DOI:** 10.3390/ani11113196

**Published:** 2021-11-09

**Authors:** Marian Czauderna, Małgorzata Białek, Edyta Molik, Kamil Zaworski

**Affiliations:** 1 The Kielanowski Institute of Animal Physiology and Nutrition, Polish Academy of Sciences, 05-110 Jabłonna, Poland; m.bialek@ifzz.pl (M.B.); k.zaworski@ifzz.pl (K.Z.); 2 Faculty of Animal Science, University of Agriculture in Cracow, 30-239 Kraków, Poland; rzmolik@cyf-kr.edu.pl

**Keywords:** orotic acid, sheep, cow, reversed-phase chromatography

## Abstract

**Simple Summary:**

Liquid chromatography for determination of orotic acid (OAc) in milk of ewes and cows is presented. Acetonitrile was added to collected milk samples; then, the resulting mixture was centrifuged. Water was added to the obtained supernatant. Finally, the obtained solution was injected into two analytical columns. All analyses were performed at a column temperature of 35 °C. Suitable separation of OAc from endogenous species of milk can be achieved using the gradient elution program and UV detection. The current chromatographic procedure resulted in satisfactory precision, accuracy and sensitivity of OAc analyses in milk samples; OAc eluted at ca. 6.4 min. Our improved chromatographic method is suitable for routine determination of OAc in milk of sheep and cows.

**Abstract:**

Ultra-fast liquid chromatography (UFLC) with a photodiode array detector (DAD) for simple and rapid determination of orotic acid (OAc) in milk of sheep and cows is described. Milk samples are treated with acetonitrile (1:1, *v/v*) and then centrifuged at 4 °C. To 1 mL of the obtained supernatant 9 mL of ultrapure water was added. Subsequently, 0.5–6 µL of the resulting solution was injected into the UFLC-DAD system. Separation and quantification of OAc in milk samples was achieved using two Kinetex C18 columns (1.7 µm, 150 mm × 2.1 mm, i.d., 100 Å; Phenomenex) fitted with a pre-column of 4 mm × 2 mm, i.d. (Phenomenex) containing C_18_ packing material. All separations were performed at a column temperature of 35 °C while the ambient temperature was 21–24 °C. Satisfactory separation of OAc from endogenous species of milk can be achieved using the binary gradient elution program and UV detection at wavelengths 278 nm. Our original procedure resulted in suitable separation and quantification of OAc in milk samples; OAc eluted at 6.44 ± 0.03 min. The total run time of OAc analysis (including re-equilibration) was 27 min. As expected, the OAc peak was absent from the blank when the proposed gradient elution program and UV detection at 278 nm was used. The average recoveries of OAc standards added to milk samples were satisfactory (96.7–105.3%). The low inter-and intra-assay coefficient of variation derived from the measurements of OAc in cow and ovine milk samples (i.e., 0.784%, 1.283% and 0.710%, 1.221%, respectively) and in O-Ac standards (i.e., 0.377% and 0.294%, respectively), as well as high recoveries of OAc added to ovine and cows’ milk (~100%) and the low detection (0.04 ng) and quantification (0.12 ng) limits point to satisfactory accuracy, precision and sensitivity of the reported method. OAc concentrations in ovine milk samples were within the range from 25 to 36 mg/L, while OAc levels in cows’ milk samples was found in the range of 32–36 mg/L. Our original procedure is suitable for routine quantification of OAc in milk of ewes and cows.

## 1. Introduction

Milk and dairy products are nutritious food products containing numerous essential nutrients such as proteins, amino acids, conjugated fatty acids, minerals (e.g., Ca, Mg, Se, Zn or I), riboflavin and vitamins (e.g., E, A, B_9_, or B_12_) [1,2]. Milk is also a good source of vitamin B_13_ (i.e., orotic acid). Orotic acid (uracil-6-carboxylic acid) was historically considered to be part of the vitamin B complex and was referred to as vitamin B_13_, but it was later discovered that it is not, in fact, a vitamin [3]. Recent studies documented that orotic acid (OAc) contributes to the non-protein nitrogen pool of ruminant milk [4]. Therefore, bovine milk is the major source of OAc in the human diet. OAc is also present at lower concentrations in the milk of other ruminants (such as goats or sheep). Cells of the mammary gland are responsible for biosynthesis and secretion of OAc into milk [4]; dihydroorotate dehydrogenase catalyses the biosynthesis of OAc. OAc is a key intermediate of de novo pyrimidine metabolism in mammalian tissues leading to the biosynthesis of cytidine, uridine, and thymidine nucleobase derivatives. Interestingly, it was discovered that OAc could be also biosynthesized by humans [3].

OAc (a heterocyclic compound) is essential for the muscular, nervous, immune, and excretory systems as well as for maintaining a healthy skin appearance [3]. OAc converts to beta-alanine, which is the precursor of anserine and carnosine. These are powerful “proton buffering agents” which delay fatigue and help boost endurance in athletes by preventing the build-up of lactic acid. OAc is very important in the biosynthesis of deoxyribonucleic (DNA) and ribonucleic acids (RNA) [3]. Some investigations show that dietary OAc can be important for patients who are at a higher risk of developing lifestyle-related cardiovascular disorders. It can boost cardiac contractility and prevent the accumulation of cholesterol plaques in blood vessels. 

Considerable variation in levels of OAc in milk can be partly explained by genetics, breed differences, lactation stage, or parity [5]. Interestingly, concentrations of OAc in milk are higher in cows, sheep, and goats (20–100 mg/L, 20–400 mg/mL, 200–400 mg/mL, respectively) compared with human milk (under 2 mg/L), sow milk (<1 mg/L), cat milk (<1 mg/L) or milk from rats (<9 mg/L) [3]. Taking into account all the above-mentioned bioactive properties of OAc it seems of utmost importance to establish a simple and fast analytical method for determining nutritionally important compounds in food products. 

So far, OAc in milk and dairy products can be determined by Fourier transform infrared spectral analysis [4,6], nuclear magnetic resonance spectroscopy [7] and ion-chromatography [8] as well as using capillary electrophoretic [9], microbiological [10], polarographic, colorimetric [11], enzymatic [12], and enzymatic-spectrophotometric [13] methods. These methods have a very wide interval concentration range (19–664 mg OAc/L) [3,4,14,15]. However, matrix effects [16] of these methods can dramatically influence analysis performance for both identification and precision of quantifications of analytes in biological samples. Moreover, these analytical procedures are expensive and/or time consuming. Considering the above-mentioned facts, it seems reasonable to improve the reversed-phase chromatographic method without derivatization for simple and rapid determination of OAc in milk of sheep and cows. The combination of an ultra-fast liquid chromatography (UFLC) with selective reversed-phase C_18_–columns and photodiode array UV-detection provides an acceptable modern analytical tool for sensitive and selective quantification of OAc in milk samples. 

## 2. Materials and Methods

### 2.1. Chemicals and Reagents

Super-gradient HPLC acetonitryl and ≥98% orotic acid (titration; anhydrous) were purchased from Sigma-Aldrich Co. (St. Louis, MO, USA). NaH_2_PO_4_∙2H_2_O and 85% ortho-phosphoric acid were of analytical reagent grade and were obtained from Avantor Performance Materials (Gliwice, Poland). Water used for the preparation of mobile phases and solutions of chemicals was purified using an Elix^TM^ water purification system (Millipore, Toronto, ON, Canada). 

Ovine milk samples were donated by the Faculty of Animal Science, University of Agriculture in Cracow (Poland). Samples of cow milk (3.2% fat) and full-fat powdered cow milk (Mlekpol, Grajewo, Poland) were purchased in local groceries in Warsaw.

### 2.2. Chromatographic Equipment, Conditions and Gradient Composition

An ultra-fast liquid chromatograph (SHIMADZU, Kyoto, Japan), incorporating two LC-20ADXR liquid chromatographic pumps (UFLCXR), a SIL-20ACXR autosampler (LFLCXR), a CBM-20A communications bus module (UFLC), a CTO-20A column oven, a DGU-20A5 degasser, and a SPD diode array detector (DAD), was used in this study. The autosampler thermostat was set to 23 °C. A sensitive DAD, was equipped with a 10 μL flow-cell. The DAD was operated in the UV range of 190–600 nm with a measurement frequency of 1 spectrum per s and spectral resolution of 1.2 nm. Two serially connected analytical Phenomenex C_18_-columns (Kinetex^®^; 1.7 μm, 100 Å, 150 mm × 2.1 mm) were used in conjunction with a guard column containing C_18_-pellicular packing material (Phenomenex C_18_; 5 mm × 4 mm); the guard column was placed in front of the analytical columns for protection. The ambient temperature was 21–24 °C, while a column heater maintained the temperature at 35 °C. The maximum system pressure was 55.5 ± 0.1 MPa.

A binary gradient elution program was used for the complete separation of OAc from background fluctuations and interfering species present in milk samples. The following elution solvents were used: solvent A was 0.02 M NaH_2_PO_4_ buffered to pH 2.2 with 10% phosphoric acid. Solvent A was filtered through a 0.2 µm membrane filter (Millipore). Solvent B was super-gradient HPLC acetonitrile. The binary gradient composition is shown in Table 1. 

### 2.3. Analytical Validation of the Method for Determination of OAc in Milk 

The limit of detection (LOD) was calculated at a signal (S_n_)—to—noise (σ) ratio of 3 (LOD = 3 × S_n_/σ), whereas the limit of quantification (LOQ) was defined as 10 times the noise under an analytical peak (LOQ = 10 × S_n_/σ); the noise (σ) under the analytical peak was calculated from the noise from the left (ơ_L_) and right (ơ_R_) side of the analytical peak (i.e., σ = (σ_L_ + σ_R_)/2) [17,18]. 

Reproducibility of the current method was also assessed by analyzing the inter- and intra-assay coefficient of variation (C.V., %) calculated from the measurements of OAc amounts in standard solutions and milk of sheep and cows. C.V., % was calculated as [19,20]:

C.V., % = (SD/µ) × 100%

where: SD is the standard of deviation of OAc measurements in assayed samples, µ is the mean value of OAc measurements in OAc standards or assayed milk of sheep and cows. 

Accuracy of the procedure was tested by adding known quantities (A) of OAc standards to milk samples and calculating the percentage recovery (R, %). Recovery was calculated as [20]:

R, % = (^F^S_n_ − ^O^S_n_) × 100%/A

where: ^O^S_n_ (ng) and ^F^S_n_ (ng) are the measurements before and after addition of OAc standard to milk samples; A—the amount (ng) of added OAc standard to milk samples. 

### 2.4. OAc Standard Sample Preparation

In the case of standard solutions of OAc, appropriate amounts (0.1–1 mg) of OAc were dissolved in 10 mL of the solution of acetonitrile and water (5:95, *v/v*). The resulting solution was vortexed, centrifuged at 15,000× *g* for 15 min (at 4 °C) and then the clear solution was transferred to a vial. 0.1–10 µL of OAc standard solutions were injected into the columns for chromatographic analysis.

### 2.5. Milk Sample Preparation

After collection, ovine milk was immediately frozen and kept in the freezer at −30 °C until analysis. Samples of fluid and full-fat powdered (FFP) cow milk were kept in original closed plastic containers at −30 °C until analysis. On the day of analysis, defrosted milk of sheep and cows were transferred to glass bottles and then warmed to 37–38 °C and briefly sonicated to achieve homogeneity of the specimen. For the same reason the sampling of milk was performed at 37–38 °C (with continuous stirring). 1 mL of milk of sheep or cows was deproteinized with 1 mL of acetonitrile, agitated for 1 min., and centrifuged at 15,000× *g* for 15 min (at 4 °C). For chromatographic analysis, 1 mL of supernatant was diluted with 9 mL of UPLC grade water. The resulting solution was vortexed and then 0.5–6 µL of the final solution were injected in to the columns. 

FFP cow milk samples (ca. 100 mg) were weighed accurately into centrifuge glass tubes and dissolved in 1 mL warm water (38 °C) and sonicated for 10–15 min to achieve homogeneity of the specimen. An amount of 1 mL of the resulting solution was treated with 1 mL of acetonitrile and centrifuged at 15,000× *g* for 15 min (at 4 °C). An amount of 1 mL of the supernatant was diluted with 9 mL of UFLC grade water. The resulting solution was vortexed and then the clear solution was transferred to a vial; 0.5–6 µL of the milk solution were injected onto the columns for chromatographic analysis.

All chromatographically analyzed OAc standard solutions and milk samples were protected from the light. The OAc peak in analyzed milk samples was identified on the basis of retention time (RT), UV spectra, and confirmed by adding OAc standard solutions to assayed milk samples. 

## 3. Results and Discussion 

**Figure 1:**
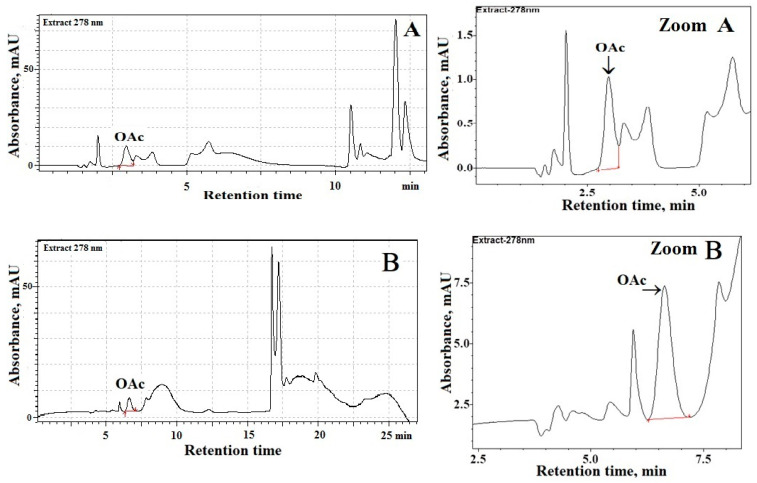

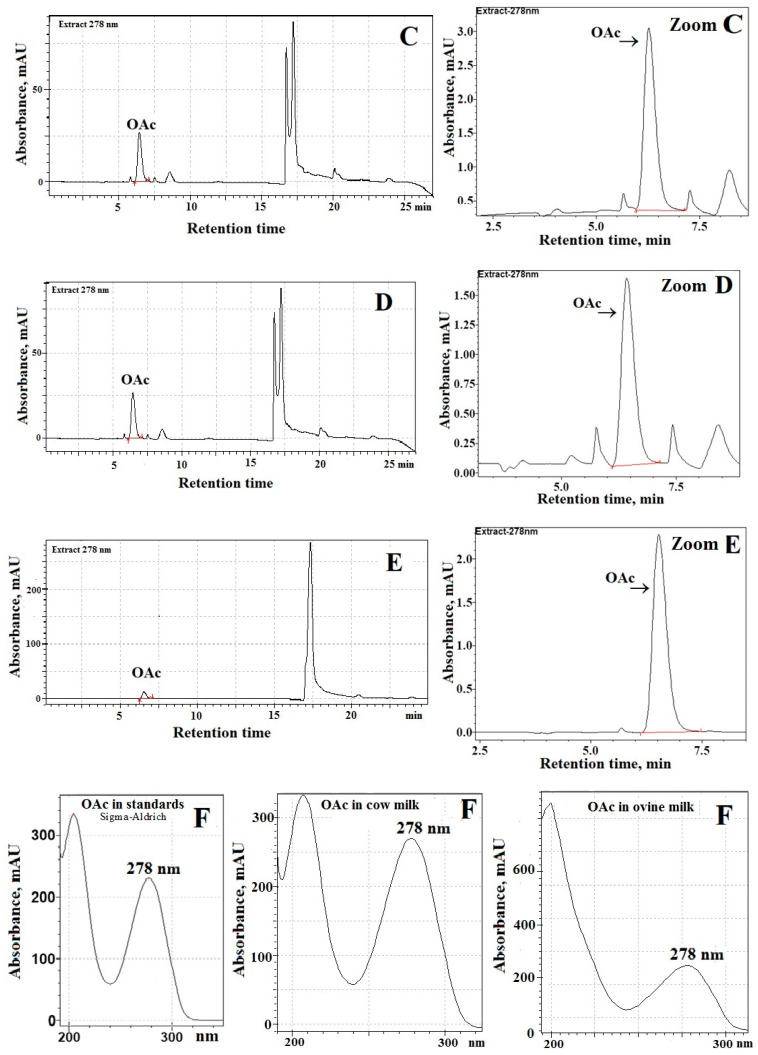
The typical chromatogram for (**A**) ovine milk samples using one analytical C_18_-column (an injection volume: 3 µL; a column temperature: 23 °C). Chromatographic separations by using two analytical C_18_-columns (injection volumes: 6 µL; a column temperature: 35 °C) for (**B**)—ovine milk, (**C**)—cow milk, (**D**)—full-fat powdered cow milk, (**E**)—OAc standard (0.015 mg/mL), (**F**)—typical stop-flow UV absorbance spectrum of OAc in analyzed standards (Sigma-Aldrich) and milk of sheep and cows.

The main analytical problem in the present study was obtaining suitable separation of OAc from the interfering compounds of milk samples. The second problem was achieving good separation of OAc without a pre-column derivatization. Therefore, in order to improve the selectivity of our chromatographic procedure, a reversed-phase C_18_-column containing dimethyloctadecylsilyl-bonded amorphous silica was used, because these columns resulted in suitable separation of analytes of even poor hydrophobicity from aqueous solutions [20,21,22]. So, we argued that OAc separation in milk samples can be achieved by using a C_18_-column. However, in milk samples, many other compounds (e.g., lactic, acetic or citric acids), similar to OAc, are poorly retained on C_18_-columns or have high absorbances. Figure 1A shows a chromatogram of processed ovine milk samples using one C_18_-column (Kinetex^®^; 1.7 μm, 100 Å, 150 mm × 2.1 mm). These chromatographic separations were performed at a column temperature of 23 °C while the ambient temperature was 21–24 °C. The use of monitoring at the maximum of UV-absorption of OAc (i.e., 278 nm; see Figure 1F) assures optimal separation of OAc from endogenous species present in the milk of sheep and cows. Chromatographic analyses (Figure 1A) evidenced that background fluctuations (a noise) and the presence of endogenous species can interfere with the accurate and precise integration of the OAc peak in sheep milk. Similarly, poor chromatographic separations of OAc from interfering species were achieved in analyzed cow milk samples (data not shown). Therefore, to avoid problems of overlapping peaks, a system of two C_18_-columns was used for OAc determination in milk samples (Figure 1B–D). Indeed, as can be seen from Figure 1B–E, the combination of chromatographic separation by using two long C_18_-columns (connected in series), UV-detection at 278 nm, and the procedure without pre-column derivatization of OAc provides a suitable analytical tool for simple and fast determination of OAc in milk samples. Moreover, satisfactory separation of OAc from endogenous species present in milk of sheep and cows was achieved due to the use of 0.02 M NaH_2_PO_4_ buffered to pH 2.2 (solvent A). Furthermore, the relatively fast elution of OAc and excellent OAc peak shapes, close to symmetrical, was observed (Figure 1B–E) due to the use of a phosphate buffer (Table 1). As a consequence, our procedure resulted in suitable separation and quantification of OAc in milk of sheep and cows, which eluted at 6.44 ± 0.03 min. Detailed chromatographic analyses documented that OAc detection at 278 nm provides the greatest response of the detector (i.e., signal (S_n_)—to—noise (σ) ratio) as compared with UV-detection of OAc in milk samples at a shorter wavelength (λ < 240 nm). As expected, the OAc peak was absent from the blank when the binary gradient elution program (Table 1) and UV-detection at 278 nm was used. 

An important analytical problem addressed in the current study was to select the optimal detection wavelength (λ_nm_) for underivatized OAc to avoid the interference of endogenous substances present in all assayed milk samples [23]. Thus, different monitoring wavelengths were applied to ensure that the species present in the assayed milk samples did not interfere with the separation efficiency of the OAc peak. Therefore, the purity (P, %) of the OAc peak in the analyzed milk samples was assessed by determining the relationships between the monitoring wavelength (λ) and the ratios (R) of the OAc peak areas (S_n_^sample^) in the milk samples (^sample^R) and comparing them to the OAc peak area (^standard^S) in the processed standards (^standard^R), i.e., P, % = (^sample^R/^standard^R) × 100% [20]. The values of ^standard^R and ^sample^R were calculated using the relationship between the OAc peak area monitored at the absorption maximum—278 nm (^max^S_n_^standard^ and ^max^S_n_^sample^, respectively; Figure 1F) and the OAc peak areas in the standard (^λ^S_n_^standard^) and milk samples (^λ^S_n_^sample^) obtained at wavelengths (λ) ranging from 190–315 nm (i.e., ^standard^R = ^λ^S_n_^standard/max^S_n_^standard^ and ^sample^R = ^λ^S_n_^sample/max^S_n_^sample^). Selected values of purity (P, %) of OAc in the analyzed milk samples are summarized in Table 2. The comparison of the obtained results indicates that in the UV range 230–295 nm the OAc peak for the assayed milk samples was pure (*P* > 90%) and free from the influence of the closely located signals of unidentified species. Moreover, the results proved that in the UV detection range 274–282 nm (Table 2) the OAc peak was the purest (*P* > 98%); therefore, we argued that UV monitoring at 278 nm ensures optimal selectivity of determination of OAc in analyzed milk samples. Really, chromatographic analyses of the assayed milk samples indicated that the OAc peak monitored at 278 nm could be most suitably integrated using the total peak area method, since this signal was devoid of the effect of substantial co-eluting impurities and endogenous species of significantly smaller absorptivity in the UV range 474–282 nm than the absorptivity of OAc in analyzed milk of sheep and cows. 

Reproducibility of the current method was assessed by performing replicate injections of processed OAc standards. Moreover, reproducibility of the present method was also assessed by analyzing the linearity of OAc quantification in standard solutions. The corresponding equations, coefficients of correlation (r) and standard error in slope are given in Table 3. Our results documented that the relationship between the OAc amounts (ng) injected into the columns and peak areas was linear over a wide range of OAc amounts in standards. Small values of the leading coefficient (2 × 10^−15^) in the quadratic equation and standard error of the slopes compared with the values of the second coefficient (7 × 10^−8^) and correlation coefficients equaling nearly 1 evidence that our new method and the proposed chromatographic elution with UV monitoring at 278 nm, provide an analytical tool of good linearity for determination of OAc in milk samples. No changes in the linearity and the detector response to 1 ng of OAc were observed when the OAc standard solutions were stored for 24 h at 4–6 °C. Moreover, in the gradient elution system developed in this study, the OAc peak in standard solutions stored for 96 h at 4–6 °C is completely separated from all background interferences. 

Table 3 summarizes the values of the limits of detection (LOD) and quantification (LOQ) in standard solutions. Chromatographic analyses showed that monitoring at 278 nm offered excellent sensitivity, as values of LOD and LOQ are very low (i.e., 0.04 and 0.12 ng).

Reproducibility of the current method was also assessed by analyzing the inter- and intra-assay coefficient of variations (C.V., %) calculated from the measurements of OAc amounts in standard solutions and milk samples (Table 4). Obtained results indicated that the proposed method offers satisfactory precision, as the inter- and intra-assay C.V., % values are low for OAc standard solutions and processed milk of sheep and cows.

Accuracy of the method was assessed by examining the recovery of known quantities of the OAc standards added to milk of sheep and cows. The results of the recovery studies are summarized in Table 5. It was found that OAc in milk of sheep and cows can be quantified reliably using the proposed method, because the obtained recoveries are satisfactory (96–106%). No changes in recoveries were observed when the processed milk samples were stored for 24 h at 4–6 °C. Moreover, our detailed study documented that the area (S_n_) of the OAc peak in analyzed milk of sheep and cows was constant for 24 h at 4–6 °C. Moreover, the chromatographic analyses showed that the OAc peak is completely separated from background interference and endogenous species present in analyzed milk samples. OAc concentrations in ovine milk samples were within the range from 25 to 36 mg/L, while OAc levels in cows’ milk samples was found in the range of 32–36 mg/L.

**Figure 2:**
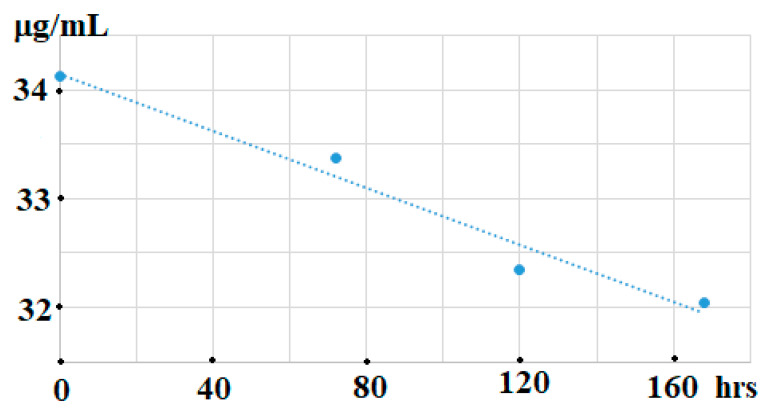
The concentrations (µg/mL) of OAc in ovine milk stored at 23 °C for 168 h (injection volumes: 6 µL).

Exhaustive investigations of the current method have demonstrated that a slight decrease of OAc contents (~6%) was observed in milk samples stored at 23 °C for 168 h (Figure 2). Fortunately, there was no overlapping of the OAc peak with background fluctuations and components present in those stored samples of milk of sheep and cows.

Our improved reversed-phase UFLC method with optimized photodiode detection to be an alternative to other analytical chromatographic methods that use very expensive ion-exchange columns [8,21,24,25]. Moreover, co-elution of other organic acids and overlapping peaks in ion-exchange columns have been frequently observed [24]. The proposed method is advantageous in some of these respects: easier manipulation of the analytical parameters to optimise the separation and the use of more inexpensive C_18_-columns [20,21,22,26]. Our chromatographic system with the very sensitive photodiode detector equipped with 10 µL of a flow cell allowed for very sensitive, as well as a relatively precise, accurate, and very simple determination of OAc in milk of ewes and cows. Ultra-fast chromatographic analyses of milk of sheep and cows showed that the peak of underivatized OAc monitored at 278 nm could be most suitably integrated, since this signal was devoid of the effect of substantial co-eluting impurities and endogenous species of significantly smaller absorptivity in the UV range of 274–282 nm than at 200–220 nm (i.e., the short-wavelength range).

Moreover, the use of the very selective two C_18_-columns and OAc monitoring at the longer wavelength (i.e., 278 nm) reduced matrix effects compared to Fourier transform infrared spectral analysis [6], nuclear magnetic resonance spectroscopy [7], as well as microbiological [10], polarographic, colorimetric [11], enzymatic [12], and enzymatic-spectrophotometric [13] methods. 

All these key features proposed that our improved UFLC method can be considered as advantageous over other analytical methods of OAc determination in milk samples.

## 4. Conclusions

Our original ultra-fast chromatographic method with optimized photodiode detection at 278 nm is a very simple analytical tool that assures rapid, accurate, and precise analyses of OAc in milk samples with extremely good determination sensitivity. The proposed method was more selective compared to chromatographic methods using one analytical C_18_-column. Therefore, the presented method based on relatively inexpensive and widely available C_18_-columns, and the very simple and rapid pre-column sample preparation method, provides the analytical procedure suitable for routine determination of OAc in milk samples.

## Figures and Tables

**Table 1:** Gradient composition ^a^.

Time (min)	Composition, % ^b^	
	Solvent A (Phosphate Buffer)	Solvent B (Acetonitrile)
0	100	0
7.8	100	0
8.0 ^c^	75	25
8.5	50	50
9.0	40	60
16.0	40	60
18 ^d^	100	0

^a^—flow-rate: 0.20 mL/min. From 0 to 7.8 min—the system pressure was 55.5 ± 0.1 MPa; from 7.8 to 16.0 min—the system pressure decreased from 55.5 ± 0.1 MPa to 47.1 ± 0.1 MPa; from 16.0 to 18.0 min—the system pressure increased from 47.1 ± 0.1 MPa to 51.7 ± 0.1 MPa; ^b^—all changes of solvent composition were linear; ^c^—the columns should be cleaned for 10 min when injected milk samples; ^d^—after 18 min, the columns was re-equilibrated for 9 min in 100% solution A (flow-rate = 0.20 mL/min).

**Table 2:** Purity (P, %) of the OAc peaks in milk of sheep and cows for particular OAc-monitoring wavelengths (λ, nm). The injection volume of processed milk samples: 6 µL.

UV Monitoring Wavelength (λ) nm	Ovine Milk ^a^ P, %	Cow Milk ^a^ P, %	FFP Cow Milk ^b^ P, %
190	64.6	81.7	74.6
200	59.6	80.8	83.0
210	75.8	85.9	84.5
220	89.1	92.4	89.3
230	93.5	96.2	94.7
240	96.5	95.9	96.1
250	93.7	94.6	94.3
260	85.2	94.3	95.8
264	87.3	95.8	96.4
268	93.8	96.4	96.7
270	97.7	96.5	97.3
272	98.5	97.8	98.6
274	99.4	98.6	99.2
276	99.8	99.7	99.7
278 ^c^	100.0	100.0	100.0
280	99.8	99.6	99.8
282	99.6	98.6	98.7
285	98.3	97.3	98.3
290	92.5	96.9	95.2
295	91.8	95.2	94.6
300	88.4	93.7	94.8
305	75.6	87.5	90.3
310	72.3	77.3	84.2
315	59.4	68.4	63.9

^a^ Purity analyses based on 1 mL of processed milk of sheep and cows; ^b^ Purity analyses based on 100 mg of processed full-fat powdered cow milk; ^c^ The absorbance maximum of UV spectrum of OAc (Figure 1F).

**Table 3:** Regression lines for the calibration curves, analysis of the linearity, standard error in slope, and the limit of detection (LOD) and quantification (LOQ) derived from determination of OAc in standards; UV detection at 278 nm.

Item	Values
Range of amount standards of OAc (ng) injected into columns	4–800 ^a^
Regression equation ^b^ Correlation coefficient (r) Standard error in slope Detector response to 1 ng OAc ^c^	y(µg) = 8.583 × 10^−8^ S_n_ − 0.0085 0.9986 1.391 × 10^−9^ 11 651
Quadratic equation Correlation coefficient (r)	y(µg) = 2 × 10^−15^ S_n_^2^ + 7 × 10^−8^ S_n_ + 0.0018 0.99987
LOD, ng	0.04
LOQ, ng	0.12

^a^ The amounts (ng) of OAc injected onto the columns: 4, 8, 20, 40, 120, 240, 400 and 800; ^b^ S_n_ and y(ng) are the OAc peak area and the amount of OAc (ng) calculated from the equation, respectively; ^c^ Detector (DAD) response at 278 nm to 1 ng of OAc in a processed standard solution.

**Table 4:** The inter- and intra-assay coefficient of variations (C.V., %) from the measurements of OAc concentrations in OAc standard solutions and milk of sheep and cows.

Item	OAc Standard	Ovine Milk	Cow Milk
The inter-assay C.V., %	0.377 ^a^	1.283 ^c^	0.784 ^c^
The intra-assay C.V., %	0.294 ^b^	1.221 ^d^	0.710 ^d^

^a^ The average inter-assay C.V., % based on the OAc standard solutions (0.04 mg/mL) repeated 7 times (preparation of seven OAc solutions each with two injections; the injection volumes: 3 and 6 µL); C.V., % = (^3µL^C.V.,% + ^6µL^C.V.,%)/2; ^b^ The average intra-assay C.V., % for repeated injections based on two OAc standard solutions (0.02 and 0.04 mg/mL) each with six injections (the injection volume: 6 µL); C.V., % = (^0.2mg/mL^C.V.,% + ^0.4mg/mL^C.V.,%)/2; ^c^ The inter-assay C.V., % based on processed 1 mL of milk of sheep and cows repeated 7 times (preparation of seven solutions of milk of sheep and cows; each milk sample was injected once; the injection volume: 6 µL); C.V., % = (SD/µ) × 100%, where: SD—the standard of deviation, µ—the mean; ^d^ The intra-assay C.V., % for repeated injections based on processed 1 mL of milk of sheep and cows; each milk sample was injected 7 times (the injection volume: 6 µL); C.V., % = (SD/µ) × 100%, where: SD—the standard of deviation, µ—the mean.

**Table 5:** Recoveries (R, %) ^a^ of OAc standards added to milk of sheep and cows and FFP cow milk.

OAc Standard Added [ng]	Recovery, %		
	Ovine Milk ^b^	Cow Milk ^b^	FFP Cow Milk ^c^
8	102.3	102.0	100.1
24	101.8	99.3	97.4
48	104.2	98.7	96.7
96	98.7	103.2	103.1
144	105.3	98.8	96.9
Pooled data	102.5 ± 2.6	100.4 ± 2.1	98.8 ± 2.8
Correlation coefficient (r) ^d^	0.9967	0.99971	0.99973

^a^ Injection volumes = 6 µL; ^b^ Processed 1 mL of milk of sheep and cows; average recoveries (R, %) based on two replications (*n* = 2); ^c^ Processed 100 mg full-fat powdered cow milk (*n* = 1); ^d^ The correlation coefficient between the contents of OAc in milk samples spiked with OAc standards (0, 8, 24, 48, 96 and 144 ng) and the detector responses (S_n_) at 278 nm.

## Data Availability

Not applicable.
